# Lithium-Associated Hypercalcemia: Pathophysiology, Prevalence, Management

**DOI:** 10.1007/s00268-017-4328-5

**Published:** 2017-12-19

**Authors:** Adrian D. Meehan, Ruzan Udumyan, Mathias Kardell, Mikael Landén, Johannes Järhult, Göran Wallin

**Affiliations:** 1 Department of Geriatrics, Faculty of Medicine and Health, Örebro University, 701 85 Örebro, Sweden; 2 Clinical Epidemiology and Biostatistics, School of Medical Sciences, Örebro University Hospital, Örebro, Sweden; 3 Section of Psychiatry and Neurochemistry, The Sahlgrenska Academy at University of Gothenburg, Sahlgrenska University Hospital, Gothenburg, Sweden; 4 Department of Surgery, Ryhov Hospital, Jönköping, Sweden; 5 Department of Surgery, Faculty of Medicine and Health, Örebro University, Örebro, Sweden

## Abstract

**Background:**

Lithium-associated hypercalcemia (LAH) is an ill-defined endocrinopathy. The aim of the present study was to determine the prevalence of hypercalcemia in a cohort of bipolar patients (BP) with and without concomitant lithium treatment and to study surgical outcomes for lithium-associated hyperparathyroidism.

**Methods:**

Retrospective data, including laboratory results, surgical outcomes and medications, were collected from 313 BP treated with lithium from two psychiatric outpatient units in central Sweden. In addition, data were collected from 148 BP without lithium and a randomly selected control population of 102 individuals. Logistic regression was used to compare odds of hypercalcemia in these respective populations.

**Results:**

The prevalence of lithium-associated hypercalcemia was 26%. Mild hypercalcemia was detected in 87 out of 563 study participants. The odds of hypercalcemia were significantly higher in BP with lithium treatment compared with BP unexposed to lithium (adjusted OR 13.45; 95% CI 3.09, 58.55; *p* = 0.001). No significant difference was detected between BP without lithium and control population (adjusted OR 2.40; 95% CI 0.38, 15.41; *p* = 0.355). Seven BP with lithium underwent surgery where an average of two parathyroid glands was removed. Parathyroid hyperplasia was present in four patients (57%) at the initial operation. One patient had persistent disease after the initial operation, and six patients had recurrent disease at follow-up time which was on average 10 years.

**Conclusion:**

The high prevalence of LAH justifies the regular monitoring of calcium homeostasis, particularly in high-risk groups. If surgery is necessary, bilateral neck exploration should be considered in patients on chronic lithium treatment. Prospective studies are needed.

## Introduction

### Historic background

Lithium has been used for a wide variety of ailments since the nineteenth century [1]. However, it was not until the illustrative case presentations of Cade [2] in 1949, and more convincingly with the work of Schou [3] in what may have been the first randomised study in psychiatry, that lithium became a more widely accepted medication in the treatment of mania in patients with bipolar disorder (BPD), previously known as manic depression. Organic disturbances associated with lithium salts were first documented by Garfinkel et al. [4] in 1973 who presented cases of both hypothyroidism and hyperparathyroidism in patients with concurrent lithium treatment. Since then, a series of articles have confirmed these initial observations. Studies concerning lithium and hypercalcemia are overwhelmingly retrospective in design and are limited in number (PubMed search with the words lithium AND hypercalcemia in January 2017 revealed only 139 articles). Lithium-associated hypercalcemia (LAH) is generally recognised as a prevailing side effect of lithium so long as treatment is necessary [5]. Lithium remains the golden standard for BPD, proving efficacious in the treatment of mania, as supplementary treatment for resistant depression and even reported as having anti-suicidal effects [6, 7]. Lithium is frequently prescribed as a chronic medication and is in most cases lifelong thus necessitating the regular monitoring of calcium homeostasis; clinically, this has not been done systematically, where the focus of regular blood work-ups has been lithium concentration, thyroid and kidney function. It has been suggested that individuals with BPD may have genetically modified calcium receptors, potentially leading to altered calcium levels [8]. Additionally, there exists controversy concerning the histopathological background and appropriate surgical strategy of lithium-treated patients who have developed biochemical signs of hyperparathyroidism [9, 10].

## Aims of the study

The aim of the present study is to determine the prevalence of hypercalcemia in bipolar patients with ongoing lithium treatment and compare this firstly to bipolar patients without lithium treatment and secondly to a randomly selected control population. A secondary aim is to study the long-term results of surgery for lithium-associated hyperparathyroidism (LHPT).

## Materials and methods

**Fig. 1:**
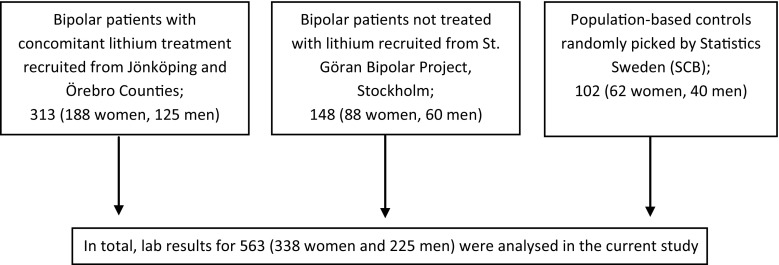
Consort diagram for patient recruitment

The patients investigated in this study come from three separate research populations (Fig. 1). The 313 bipolar patients with ongoing lithium treatment were recruited as part of a prevalence study conducted between October 2012 and March 2014. These patients attended one of the affective outpatient units in either Jönköping County or Örebro County, Sweden. These two socio-economically similar counties, located in central Sweden, have about 460,000 residents, which is approximately 4.5% of the Swedish population. Patients’ medical records were examined for all relevant information pertaining to parathyroid pathology including diagnoses, biochemistry, any possible surgery, and ongoing medication. All operations were performed by experienced endocrine surgeons. The choice and extent of surgery was at the surgeon’s discretion since no uniform policy exists.

The second population, bipolar disorder without concomitant lithium treatment, were recruited from a research project called the St. Göran’s Bipolar Project (SBP) led by one of the authors (ML) [11]. The project aims at further understanding the neurobiological mechanisms of mood disorders and started in October 2005. Regular follow-ups occur annually. Individuals were recruited from the bipolar outpatient unit at the Northern Stockholm Psychiatric Clinic, Stockholm, Sweden, with a catchment area of approximately 300,000 residents. According to available records, none of these patients have previously medicated with lithium.

A third population-based control group was randomly selected by Statistics Sweden (SCB) [12]. Individuals were contacted by post and fourteen per cent of those contacted agreed to participate as volunteers, and this level of participation is, according to SCB, comparable to similar studies. For these two latter populations, variables such as biochemical status pertinent to calcium homeostasis, relevant details about somatic disease and current medication were retrieved from the SBP study’s database.

### Assessment of hypercalcemia

Data available for the assessment of hypercalcemia included total and corrected total calcium, creatinine and, where possible, parathormone. Parathormone was only available for bipolar patients *with* lithium (Table 1). Vitamin-D status was available in so few that comparison was not viable, and therefore abandoned. In addition, current medication was noted and classified according to whether the substance was a mood-stabiliser, central stimulants, antidepressant, anxiolytic, antipsychotic, anxiolytic other than benzodiazepines, and “other” medications most often pertaining to somatic disorders such as hypertension. Hypercalcemia was defined as average total calcium, TCa ≥ 2.50 mmol/l according to national guidelines.

**Table 1:** Demographic and clinical variables, including the use of psychiatric and non-psychiatric medications, of study groups

Variables	Bipolar with lithium (*n* = 313)	Bipolar without lithium (*n* = 137)	Control population (*n* = 102)	Total population (*n* = 552)	*p* value*
Female, *n* (%)	188 (60)	88 (60)	62 (61)	338 (60)	0.989
Age, median years (range)	57 (19–92)	35 (18–74)	34 (21–75)	48 (18–92)	≤0.001
Calcium mmol/l, median (range) (ref: 2.10–2.50)	2.43 (2.17–2.86)	2.29 (2.05–2.53)	2.26 (2.05–2.56)	2.38 (2.05–2.86)	≤0.001
PTH ng/l, median (range) (ref: 10–73)^€^	65 (22–305)	n/a^§^	n/a	–	–
Creatinine µmol/l, median (range) (ref: ♀ 45–90, ♂ 60–105)	77 (46–139)	67 (39–140)	69 (45–100)	73 (39–140)	≤0.001
Hypercalcemia, ≥ 2 episodes of 2.50 mmol/l (% of subsample)	82 (26.2)	2 (1.4)	3 (2.9)	87 (15.5)	≤0.001
Pathological TSH, *n* (%)	115 (36.7)	27 (19.7)	5 (4.9)	147 (26.6)	≤0.001
GAF-symptom scale, median (range)^#^	65 (25–97)	65 (45–85)	80 (60–92)	70 (25–97)	≤0.001
Duration of mood-stabilizing therapy, median years (range)^¤^	14 (2–44)	12 (1–33)	–	–	0.115
Use of medications					
Levothyroxine, *n* (%)	89 (28.4)	9 (6.6)	1 (1.0)	99 (17.9)	≤0.001
Antidepressants, *n* (%)	142 (45.4)	39 (28.5)	1 (1.0)	182 (33.0)	≤0.001
Antipsychotics, *n* (%)	105 (33.5)	27 (19.7)	0 (0.0)	132 (23.9)	≤0.001
Anxiolytics, *n* (%)	49 (15.7)	22 (16.1)	0 (0.0)	71 (12.9)	≤0.001
“Non-psychiatric” medications, *n* (%)	207 (66.1)	47 (34.3)	41 (40.2)	295 (53.4)	≤0.001

* *p* values are from a Chi-square test for categorical variables and from a median test for continuous variables

^€^Values available for 234 participants

^§^n/a = not available

^#^Values were available for 403 participants in this analysis

^¤^Information was available for 176 participants (39%): 100 participants with BPD with lithium and 76 without lithium

### Statistical analysis

Descriptive statistics included frequencies (percentages) and median values (ranges). Individuals with hypercalcemia were compared with those without hypercalcemia using Chi-square test (for categorical measures), and a nonparametric test on the equality of medians (for non-normally distributed continuous measures). The same tests were used to compare the distribution of the study covariates by the study groups (BP with lithium, BP without lithium and the control population). Unadjusted and adjusted logistic regression models compared odds of hypercalcemia in bipolar patients with ongoing lithium treatment as well as in a randomly selected control population with the corresponding odds in bipolar patients without lithium treatment.

The form of the relationship between the log odds of the hypercalcemia and continuous measures was explored using locally weighted scatterplot smoothing (LOWESS) methods, restricted cubic splines, as well as multivariable fractional polynomial methods. This analysis indicated a linear relationship with the log odds of hypercalcemia for age, GAF symptom, GAF function and creatinine. The statistical software used was Stata version 12/SE for Windows (StataCorp, College Station, Texas). Tests were two-sided, and statistical significance was defined as *p* < 0.05 and 95% confidence intervals that do not include 1.00.

### Ethics

The approval for the collection of medical data and the completion of this study was given by the Central Ethics Review Board at the University of Uppsala, Sweden (Dnr 2011/428).

## Results

**Fig. 2:**
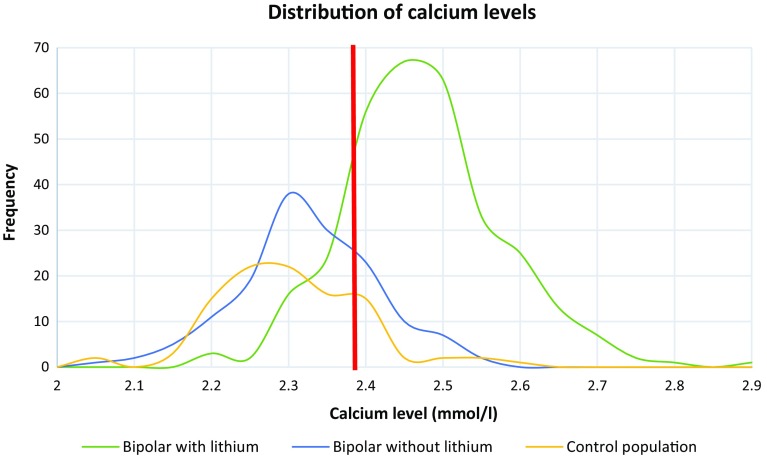
Distribution of calcium values for the three separate study groups. The available values included bipolar with lithium treatment (*n* = 313), bipolar without lithium (*n* = 148), population-based control group (*n* = 102). The median calcium value for the group as a whole was 2.37 mmol/l (illustrated with red line). Eighty-seven patients had P–Ca > 2.5 mmol/l; of those, eighty-two (94.3%) were bipolar patients with lithium treatment

The study population comprised 563 individuals with measured total calcium (Fig. 1). In comparison with bipolar *without* lithium (BWOL), the group bipolar with lithium (BWL) had statistically higher values of calcium, with a median TCa = 2.43 mmol/l (range = 2.17–2.86) (Table 1, Fig. 2). PTH was only available for BWL with a median of 65 ng/l (22–305) being reported. The prevalence of hypercalcemia was 15.8% in the whole group, though 26% in BWL. In addition, a disproportionate and significant proportion of BWL had had pathological thyroid values (*p* = ≤0.001) and medicated with levothyroxine (*p* = ≤0.001). The use of antidepressants, antipsychotics, sedatives and other “non-psychiatric” medications was more common in the BWL group (Table 1). In the group identified with hypercalcemia (Table 2), 94.3% were BWL, 67% were female and had a median age of 64 years. Concomitant lithium treatment (adjusted OR 13.45; 95% CI 3.09, 58.55; *p* = 0.001) as well as older age and gender was associated with increased odds of hypercalcemia, whereas antidepressant use was associated with reduced odds (Table 3). BWL and BWOL scored lower values in the GAF instrument but in neither the unadjusted (Table 2) nor the adjusted analysis (OR 1.01 (95% CI 0.98, 1.03)) was the GAF instrument associated with hypercalcemia (in a subgroup analysis of 403 people).

**Table 2:** Characteristics of study population according to calcium homeostasis

Variables	Hypercalcemia (*n* = 87)	Normocalcemic (*n* = 465)	Total population (*n* = 552)	*p* value*
Study groups				
Bipolar with lithium, *n* (%)	82 (94.3)	231 (49.7)	313 (56.7)	≤0.001
Bipolar without lithium, *n* (%)	2 (2.3)	135 (29.0)	137 (24.8)	
Control population, *n* (%)	3 (3.4)	99 (21.3)	102 (18.5)	
Female, *n* (%)	67 (77.0)	266 (57.2)	333 (60.3)	≤0.001
Age, median years (range)	64 (24–90)	45 (18–92)	47 (18–92)	≤0.001
Calcium mmol/l, median (range) (ref: 2.10–2.50)	2.57 (2.50–2.86)	2.36 (2.05–2.50)	2.38 (2.05–2.86)	≤0.001
Creatinine µmol/l, median (range) (ref: ♀ 45–90, ♂ 60–105)	78 (45–130)	72 (39–140)	73 (39–140)	0.161
Pathological TSH, *n* (%)	42 (48.3)	105 (22.6)	147 (26.6)	≤0.001
GAF-symptom scale, median (IQR)^#^	68 (53–75)	70 (60–80)	70 (60–78)	0.245
Use of medications				
Levothyroxine, *n* (%)	35 (40.2)	64 (13.8)	99 (17.9)	≤0.001
Antidepressants, *n* (%)	25 (28.7)	157 (33.8)	182 (33.0)	0.360
Antipsychotics, *n* (%)	34 (39.1)	98 (21.1)	132 (23.9)	≤0.001
Anxiolytics, *n* (%)	17 (19.5)	54 (11.6)	71 (12.9)	0.043
“Non-psychiatric” medications, *n* (%)	66 (75.9)	229 (49.2)	295 (53.4)	≤0.001

* *p* values are from a Chi-square test for categorical variables and from a median test for continuous variables

^#^Values were available for 403 participants in this analysis

**Table 3:** Multivariable analysis comparing hypercalcemia in bipolar patients without concomitant lithium treatment with bipolar patients with lithium and to a control population

	Unadjusted OR (95% CI)	*p* value	Adjusted OR (95% CI)	*p* value
Study groups^a^				
Bipolar without lithium	1 (ref)		1 (ref)	
Bipolar with lithium	23.96 (5.80, 99.00)	≤0.001	13.45 (3.09, 58.55)	0.001
Control population	2.05 (0.36, 12.47)	0.438	2.40 (0.38, 15.41)	0.355
Age	–		1.04 (1.02, 1.06)	≤0.001
Sex	–		0.38 (0.19, 0.77)	0.007
Creatinine	–		1.01 (0.99, 1.03)	0.489
Pathological TSH	–		1.30 (0.63, 2.69)	0.477
Levothyroxine	–		1.03 (0.46, 2.28)	0.945
Antidepressants	–		0.38 (0.21, 0.67)	0.001
Antipsychotics	–		1.88 (1.04, 3.04)	0.036
Anxiolytics	–		1.66 (0.80, 3.45)	0.174
“Non-psychiatric” medications	–		1.45 (0.75, 2.79)	0.273

^a^Complete values were available for 552 individuals

Bilateral neck exploration was performed in seven surgical cases (Table 4). Four women and three men were operated with a mean age of 60 years (range 46–74 years) after having medicated with lithium for 25 years (range 14–36 years). The histopathological diagnosis was hyperplasia in four patients at the initial operation with an average of two glands removed. One patient (case 5) with lipoadenoma had persistent hypercalcemia post-operatively, was later re-operated with extirpation of a further two parathyroid glands and was normocalcemic at latest follow-up. All other patients had recurrent disease. The average follow-up was 10 years (range 5–12 years).

**Table 4:** Demographic data, pre- and post-operative laboratory data including histopathological diagnoses and follow-up results of seven lithium-treated bipolar patients having undergone parathyroidectomy for hyperparathyroidism

Case no.	Sex	Age	Lithium duration at operation	No. glands identified	No. glands extirpated	Morphology	PTH before surgery (ref: 10–73)	Phosphate before surgery (ref: ♀ 0.8–1.5, ♂ 0.7–1.6)	Ca (ref: 2.10–2.50)		Crea (ref: ♀ 45–90, ♂ 60–105)		Thyroid disease	Cure at initial operation	Follow-up
									Before	After	Before	After			
1	F	65	28	3	1.5	Normal	106	0.99	2.73	2.47	92	77	Nontoxic goitre	No	12 years, recurrent
2	F	74	34	4	3	Hyperplasia	170	0.92	2.79	2.45	78	69	Multinodular goitre	No	7 years, recurrent
3	F	65	33	2	2	Hyperplasia	117	1.08	2.66	2.43	74	72	None	No	12 years, recurrent
4	M	50	14	4	2	Hyperplasia	67	n/a	2.75	2.02	109	109	Hypothyroidism	No	12 years, recurrent
5	M	55	29	2	1	Lipoadenoma	268	0.8	2.83	2.18	154	187	None	No	11 years, persistent^a^
6	M	62	36	2	1	Adenoma	102	n/a	2.69	2.47	107	111	Hypothyroidism	No	5 years, recurrent
7	F	46	19	4	2.5	Hyperplasia	66	n/a	2.67	2.32	81	97	Hypothyroidism	No	11 years, recurrent

^a^Patient re-operated 2016 with the extirpation of a further two parathyroid glands, weight 1820 mg; normocalcemic at latest follow-up

## Discussion

We studied hypercalcemia in bipolar disorder. The prevalence of hypercalcemia in the entire study population is 15.5%, with hypercalcemia remaining strongly associated with lithium treatment, even when adjusted for sociodemographic factors and medications (Table 3). This study demonstrates that a causal relationship between lithium treatment and the development of hypercalcemia may be presumed, though amid the plethora of possible mechanisms of action, the exact mechanism in the individual is difficult to ascertain. While polypharmacy, which was higher in the bipolar patients with lithium, may at the clinical level have bearing on the occurrence of hypercalcemia, it does not explain the statistically higher levels of calcium at group level. In addition, all seven lithium-treated bipolar patients having undergone parathyroidectomy for LAH were not cured at the initial operation; one patient had persistent disease and six patients showed clear biochemical signs of recurrent disease at follow-up (Table 4).

### How common is LAH?

Primary hyperparathyroidism is the third most common endocrine condition with a prevalence of approximately 0.5% in the general population, rising to 2–3% in postmenopausal women [12]. LAH is characterised by mildly elevated calcium levels (Fig. 2), which occur chronically or intermittently [13, 14]. Despite numerous articles highlighting the credible association between lithium and the development of hypercalcemia, the monitoring of parathyroid function has only recently been included in the revised recommendations of NICE [15] and the International Society for Bipolar Disorder [16]. In the light of this paucity of clear national and international guidelines, monitoring of parathyroid function has in many cases been scarce and irregular. This has led to evident difficulties in determining a reliable incidence and prevalence of LAH. McKnight et al. [17] reveal the absolute risk of developing LHPT is 10%, considerably higher than pHPT. Estimations of LAH or LHPT prevalence vary widely from 4.3 to 80%, though this possibly reflects the dimensions of study populations [18–21]. The present study confirms previous observations that at least a quarter of lithium-treated patients have repeated disturbances in calcium homeostasis (Table 1).

### Pathological background to hypercalcemia during lithium-treatment

Lithium is a mood stabilizer but the exact biochemical mechanism of action has yet to be fully elucidated. The lithium ion is thought to be active, particularly in signal transduction pathways at multiple sites. Two main pathways of mood-stabilizing action are proposed, namely the inhibition of inositol monophosphatase, IMPase (important for cell regulatory functions such as cell growth and apoptosis), and the inhibition glycogen synthase kinase, GSK-3 (important for apoptosis) [22–24]. By inhibiting GSK-3, long-term plasticity and neurocellular protection and stability may be achieved [25].

These mechanisms may be involved in lithium’s implicatory role in the development of hypercalcemia. Szalat et al. [26] explains lithium’s involvement in the cascade leading to the inhibition of IMPase in parathyroid chief cells, resulting in intracellular changes in calcium levels and thereby parathyroid hormone (PTH) secretion. A widely accepted theory is that lithium interacts with the calcium-sensing receptor (CaSR), a ubiquitously expressed transmembrane G-protein-coupled cell surface receptor, which in turn leads to a so-called right-shift or set-point elevation of calcium concentrations in relation to PTH [27, 28]. Chief cells then react as if the extracellular concentration of calcium has decreased. Exactly how lithium interacts with CaSR is difficult to determine. Furthermore, increased direct secretion of PTH is thought to occur through the development of adenoma or hyperplasia; hypocalciuria likely arises through the inhibition of renal cyclic adenosine monophosphate (cAMP) [13, 29].

Lithium affects PTH secretion and thereby causes PTH-dependent hypercalcemia, whereas the use of drugs such as thiazides (not uncommon in the elderly) and vitamin D can cause PTH-independent hypercalcemia.

The pathoanatomical diagnosis of LAH is a matter of some contention. Primary HPT is caused by a single adenoma in 85% of cases, with hyperplasia in 10–15% [30]. Lithium, through GSK-3 inhibition, contributes to irregular Wnt/β-catenin signalling, believed to be significant for the development of parathyroid adenomas and hyperplasia [31]. Theoretically, lithium should exert an equal effect on all parathyroid glands [32]. Carchman et al. [9], however, state that there is no significantly increased risk for multiglandular disease (MGD) in lithium-treated patients. On the other hand, numerous studies present higher frequencies of MGD ranging from 25 to 83% [10, 26, 33–37]. The surgical results in the present study further confirm these latter results with MGD occurring in five of seven patients (70%) (Table 4). This has clear ramifications for the treatment and management of LAH.

### Management of LAH

Four main strategies for management of LAH are available. Firstly, lithium may be discontinued, with calcium normalisation occurring in many though far from all cases [5]. Discontinuation may be difficult due to the serious risk of psychiatric deterioration. Secondly, careful surveillance may be an option, though the patient’s clinical status must be observed vigilantly. Thirdly, calcimimetic therapy has been described as possible strategy for LHPT, especially if continued lithium treatment is necessary [38]. This strategy is even proposed by Szalat et al. [26], particularly for those not suitable for surgery. The fourth and final strategy is surgery, though debate exists as to the degree of radicality that should be executed [9, 10, 33–37].

Surgical management of LAH is influenced by whether the condition is considered to be primarily a single glandular disease (SGD) or multiglandular (MGD). Hitherto, studies give differing views as to whether bilateral neck exploration (BNE) or focused neck exploration (FNE) is the appropriate surgical strategy. The role of pre-operative localisation techniques, has not been conclusively established for patients with LHPT [39]. Lithium is considered an exacerbating factor that “de-masks” the predisposition for the development of an adenoma, and FNE is recommended [9, 40, 41]. Awad et al. [40], in a study population of 15, found only one patient with MGD. The authors of this study (ADM, GW, JJ), in material yet unpublished, have found that four (1.5%) of 297 patients had elevated calcium levels (> 2.5 mmol/l) before the initiation of lithium treatment. The possibility of two coexisting phenomena, i.e., lithium treatment and pHPT, without any causal relationship, has to be considered [42]. Nevertheless, most studies suggest the frequent occurrence of MGD [10, 26, 34–36]. Hundley et al. [10] further point out that the resection of a single gland increases the risk of disease recurrence. On the other hand, the risk of permanent hypoparathyroidism with more radical surgery, though low, is not negligible [37]. The present recommendations of the European Society of Endocrine Surgeons (ESES) suggest that a history of lithium treatment is a relative indication for BNE [29].

### What is the significance of the present findings?

There currently exists a lack of systemization in the monitoring of parathyroid function in lithium-treated patients, which may reflect the lack of clear guidelines on both the national and international level. Regular monitoring, involving TCa, PTH and vitamin D, would potentially identify all patients with tendencies towards hypercalcemia, whereby appropriate action could be taken. Hypercalcemia in lithium-treated patients with BPD was not correlated with kidney function, and even moderate chronic kidney insufficiency proved to be statistically insignificant (Table 3). The significance of hypercalcemia for the lithium-treated individual is unclear [14]. The GAF instrument did indicate that individuals with lithium treatment scored lower on this assessment of psychosocial functioning (Tables 1, 2). It illustrates the difficulty in discerning and differentiating symptoms primarily related to bipolar disorder and those associated with hypercalcemia. No robust instruments exist to differentiate these two conditions, but the need is apparent. It is often thought that non-lithium-treated patients with mildly elevated calcium are “asymptomatic”, often leading to “doctor’s delay” [19]. Talpos et al. [43], using SF-36, reported dramatic improvements in quality of life for seemingly asymptomatic HPT patients undergoing parathyroidectomy, effects that remained 2 years post-operatively. This underlines the need to penetrate the patient’s anamnesis in a structured way in that it may play an important role in deciphering indications for surgery for LAH patients.

A large percentage of lithium-treated patients may be at risk of developing hypercalcemia, but far from all [31]. Some patients can medicate with lithium for decades without, at any time, showing tendencies towards calcium elevation. The reason for this is unclear. In the present study, lithium treatment, age, gender and thyroid disease were strongly associated with the development of hypercalcemia, while studies even point to treatment duration and dosage as associated risk factors [5, 14, 18, 28]. These factors should be considered in any assessment for surgery as they may prove important in deciding the extent of any eventual operation.

The question of surgical management of LAH needs to be explored further. Most cases presented here had MGD (Table 4), though on average only two parathyroid glands were removed. The extent of surgery at the initial operation may have importance for the risk of recurrence, particularly for patients continuing lithium medication. There exists a strong need for prospective randomized surgical studies to evaluate appropriate management strategies in relation to patient benefit. Prospective therapeutic studies are also needed, including the evaluation of calcimimetics.

### Study limitations

This study has several limitations. It is cross-sectional and thereby bereft of information as to disease debut or how calcium homeostasis changes over time. Secondly, it is retrospective, which adds to the risk that the collected information is selective and incomplete. Thirdly, though we recommend monitoring PTH and vitamin D, we have not been able to collect PTH for all the subgroups and vitamin D for only a small number of individuals. Collection of this data would have strengthened any conclusions that are made.

## Conclusion

Our study reveals that hypercalcemia is not associated with bipolar disorder but is a common, though underestimated, characteristic of lithium treatment. We believe this condition to be far more complicated than has been described earlier. We postulate that genetic factors, lithium duration and dosage, gender, other medications and pathologies may together or separately contribute to the development of this highly debated endocrinopathy. Bilateral neck exploration should be considered for those individuals able to undergo surgery.

## Abbreviations

BNE: Bilateral neck exploration
BPD: Bipolar disorder
cAMP: Cyclic adenosine monophosphate
CaSR: Calcium-sensitive receptors
FNE: Focused neck exploration
GAF: Global assessment of function
HPT: Hyperparathyroidism
iCa: Ionised calcium
LAH: Lithium-associated hypercalcemia
LHPT: Lithium-associated hyperparathyroidism
MGD: Multiglandular disease
pHPT: Primary (sporadic) hyperparathyroidism
PTH: Parathyroid hormone/parathormone
SBP: St. Göran’s Bipolar Project
SGD: Single gland disease
TCa: Total calcium
TCa_corr_: “Corrected” total calcium
